# Supplementary material for: Chromothripsis during telomere crisis is independent of NHEJ, and consistent with a replicative origin
Source: Genome Res. 2019 May;29(5):737–49. doi: 10.1101/gr.240705.118 (PMC6499312; doi:10.1101/gr.240705.118)
Supplement: Supplemental Material [file supp_gr.240705.118_Supplemental_file_1.zip › contigs/annotated_contigs/DB116/contig.2.DB116_length_687_mean_cov_11.3508005822.docx]

**DB116_length_687_mean_cov_11.3508005822**

```
ACCTTCTTATTTTAAGCTACATGGGAATTTTTTCATTTTCTTTCTTTCTTTTTTTTTTGAGACGGAGTCTTGCTCTCTCACCCAGGCTG
 >chr2:209855761-209856109 + E=1e-197 p=1e-02
GACTGCAGTGGCGTGATCTCGGCCCACTGCAAGCTCCTCCTCCTGGGTTCACGCCATTCTGCTGCTCAGCCTCCCCAGCAGCTTGAACT

ACAGGCGCACGCCGCCACGCCCGGCTAATTTTTTTGTATTTTTAGTAGAGACGGGGTTTCACCGTGTTAGCCAGGATGGTCTCGATCTC

CTGACCTCATGATCCGCCCACCTCGGCCTCCCAAAGTGCCGAGATTACAGGCGTGAGCCACCACGCCCGGCTGATTTTT|TC|TACTTA
                                                                                >chr2:2
CCATGAGCCCTCTATGAGGTCCAAGCTATTTCCAAGATGCACAAGGCATATCTGGCTCCAGGACGATTAAGGAAATAGAAATGCTGACA
09857107-209857448 + E=2e-190
AATAATCTAAGTAATCATTGAGTCAAAGAAGAAATCTGAAGATAAATCAGAAAATATTTTAAACTAAATGATAATGAAAATATGGCAGA

TCAATAATCATAGGAGTAGCTAAAGCAGTGCTTAGAAGAAAATTTATAGCGCTAAATGCCTATATTAGAACAGATAGTAACCTAACAGC

GATCTGTGGCTGCAATGTAAGAAAATAAAAGAGCAAAATAAACTCAAAGCAAACAGAAGAACAAAA
```
